# Natural *Plasmodium inui* Infections in Humans and *Anopheles cracens* Mosquito, Malaysia

**DOI:** 10.3201/eid2710.210412

**Published:** 2021-10

**Authors:** Jonathan W.K. Liew, Fatma D. Mohd Bukhari, Nantha Kumar Jeyaprakasam, Wei Kit Phang, Indra Vythilingam, Yee Ling Lau

**Affiliations:** Universiti Malaya, Kuala Lumpur, Malaysia

**Keywords:** *Plasmodium inui*, *Anopheles*, vector, malaria, humans, monkey, vector-borne infections, parasites, zoonoses, Malaysia

## Abstract

We detected 2 natural, asymptomatic *Plasmodium inui* monoinfections in humans in Malaysia by using nested PCR on concentrated high-volume blood samples. We found a *P. inui*–positive *Anopheles cracens* mosquito in the same site as the human infections. Investigators should use ultrasensitive detection methods to identify simian malaria parasite transmission in humans.

Zoonotic transmission of simian malaria parasites to humans have been occurring in Southeast Asia and South America. Among the 3 simian malaria parasites in Southeast Asia experimentally shown to infect humans (*1*), *Plasmodium knowlesi*, *P. cynomolgi*, and *P. inui*, only *P.* *knowlesi* and *P. cynomolgi* have been reported in cases of natural infection (*2*). We report 2 natural, asymptomatic *P. inui* human infections detected by using nested PCR (nPCR) on concentrated high-volume blood.

## The Study

We conducted an epidemiologic and entomological study at a campsite in Kem Sri Gading, Pahang, Malaysia (3°45′46.24″N, 102°34′20.32″E), because of frequent reports of human *P. knowlesi* infections acquired from this area. Kem Sri Gading is a receptive area, a location in which the ecosystem permits malaria transmission because vector and reservoir host populations both inhabit it. 

On March 2, 2020, we obtained <3 mL of venous blood from 71 persons at the camp who provided consent. Participants had undergone training in the forest at Kem Sri Gading during January 27–28, 2020. The Medical Research and Ethics Committee, Ministry of Health Malaysia, approved this study (approval no. NMRR-15-672-23975 for the human study and approval no. NMRR-19-962-47606 for the mosquito study).

The 2 case-patients we report, PMAR0041, a 20-year-old woman, and PMAR0052, a 19-year-old woman, had no previous history of malaria. Before our study, PMAR0041 was in a nonreceptive city in Selangor 1–2 weeks before training at the camp; PMAR0052 regularly entered forested areas >2 times per month. During January 29–March 2, 2020, neither case-patient visited any potentially receptive areas. Both persons reported they were healthy before, during, and after blood collection.

**Figure 1:**
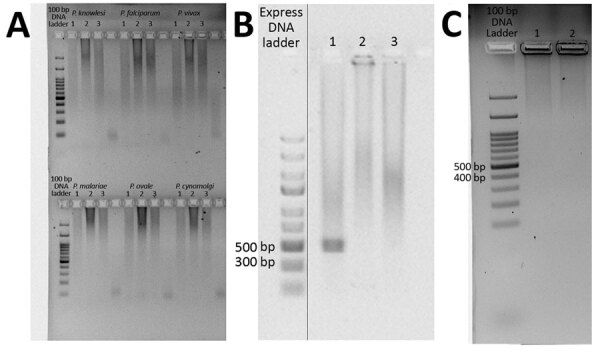
Species-specific nested PCR amplification products for a study of *Plasmodium inui* infections among humans, Malaysia. Samples were subjected to electrophoresis on a 1.5% agarose gel. A) Results for detection of *P. knowlesi*, *P. falciparum*, *P. vivax*, *P. malariae*, *P. ovale*, and *P. cynomolgi*. Lane 1, human case-patient PMAR0041; lane 2, human case-patient PMAR0052; lane 3, no-template control. B) Results for the detection of *P. inui* in human case-patient PMAR0041. Lane 1, case-patient PMAR0041; lane 2, negative control; lane 3, no-template control. The solid vertical line indicates these are separate parts of the same image. C) Results for the detection of *P. inui* in human case-patient PMAR0052. Lane 1, case-patient PMAR0052; lane 2, no-template control.

**Figure 2:**
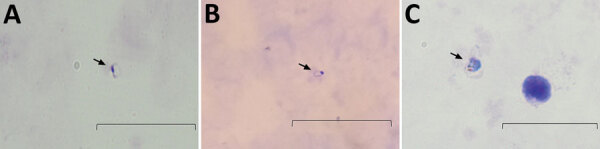
Micrographs of the thick blood smears showing *Plasmodium* trophozoites in 2 human cases of *Plamodium inui* infection, Malaysia. A) Smear from case-patient PMAR0041, taken by using an Olympus BX51 microscope (Olympus Corporation, https://www.olympus-lifescience.com). B) Smear from case-patient PMAR0041, taken by using Redmi Note 4 (Xiaomi Corporation, https://www.mi.com) smartphone camera. C) Smear from case-patient PMAR0052, taken by using an Olympus BX51 microscope. Arrows indicate *P. inui* trophozoites in each image. Scale bars indicate 20 µm.

Using the amount of DNA equivalent to 500 µL of whole blood (*3*), we detected *Plasmodium* in the 2 cases in separate nPCR assays (Appendix). We used primers targeting both the asexual and sexual 18S rRNA genes of *Plasmodium* (*4*). Sequence analysis of the cloned genus PCR products confirmed *P. inui* (Table). We performed species-specific nPCR assays to detect 5 known human malaria parasites, including *P. knowlesi*, and to detect *P. cynomolgi* and *P. inui*, by using previously published primers (*4*–*6*). However, the species-specific PCR amplification demonstrated spurious results; we were unable to produce consistent results over repeated tests. Thus, *P. inui* was detected only in case-patient PMAR0041 (Figure 1) because the protocol produced insufficient DNA, which hampered further analyses. However, we found likely trophozoites in thick blood smears of each case during 2 hours of observation (Figure 2).

**Table:** Nucleotide BLAST results of the PCR products sequenced in a study of natural transmission of *Plasmodium inui* in 2 humans and in *Anopheles cracens* mosquitoes, Malaysia*

Sequence source and length, bp (GenBank accession no.)	Description of sequence (GenBank accession no.)	% Identity	% Query cover
Patient PMAR0041, 234 (MW555281)	*Plasmodium inui* asexual type 18S rRNA, Celebes (AB287276)	99.57†	100
	*P. inui* asexual type, 18S rRNA, Thailand (EU400385)	99.57	100
	*P. inui* asexual type, 18S rRNA, Taiwan I (FN430724)	99.57	100
Patient PMAR0052, 243 (MW555282)	*P. inui* sexual type, 18S rRNA, Taiwan I (FN429982)	99.59†	100
	*P. inui* 18S rRNA, *Anopheles latens* mosquito, Sarawak (MN535358)	99.18‡	100
	*P. inui* sexual type, 18S rRNA from monkey (FJ619103)	99.18	100
	*P. inui* 18S rRNA, wild monkey, Thailand (EU400386)	99.18	100
*An. cracens*, 986 (MW555286)	*P. inui* asexual type, 18S rRNA, Celebes (AB287276)	99.90†	100
	*P. inui* 18S rRNA, *An. latens*, Sarawak (MN535320)	99.80‡	100
	*P. inui* asexual type 18S rRNA, wild monkey, Thailand (EU400385)	99.70	100
	*P. inui* asexual type 18S rRNA, South China (HM032051)	99.49	100
	*P. inui* asexual type 18S rRNA, Taiwan II (FN430725)	99.49	100


*BLAST, https://blast.ncbi.nlm.nih.gov. bp, base pair. †This sequence had only a 1 single-nucleotide mismatch at the forward primer priming site. ‡This sequence had 2 single-nucleotide mismatches, 1 at the forward primer priming site.

On October 9, 2020, we obtained a second blood sample from case-patient PMAR0041; case-patient PMAR0052 did not consent to a second blood collection. Between the first and follow-up blood collections, PMAR0041 did not travel to any receptive areas. We did not detect any *Plasmodium* DNA in the second blood sample from PMAR0041 after repeated tests.

We collected *Anopheles cracens*, *An. introlatus*, and *An. barbirostris* sensu lato mosquitoes at the camp by using human landing catches and Mosquito Magnet Independence Trap (Woodstream Corp., https://www.woodstream.com). *An. cracens* was the predominant mosquito species collected. Only 1 nonblood fed *An. cracens* mosquito, caught on August 24, 2020, was *Plasmodium*-positive in its head and thorax by nPCR (*4*). We found no oocysts upon dissection of the mosquito gut. We were unable to successfully dissect the salivary glands because the mosquito was dead. We used published primers (*7*) to amplify the *P. inui* 18S rRNA gene and confirmed *P. inui* by sequencing the PCR product (Table). We tested the entomological team by using the same PCR methods described for the case-patients but detected no *Plasmodium*.

Our analyses showed that the *P. inui* sequence obtained from case-patient PMAR0041 was identical to the corresponding region on the asexual type 18S rRNA sequence obtained from the *An. cracens* mosquito (Appendix Figure 2), but the *P. inui* sequence obtained from case-patient PMAR0052 was of the sexual type 18S rRNA (Table). The human *P. inui*–positive cases we detected originated from separate DNA extractions and PCR assays on different days by using dedicated benchtops for different procedures. The case-patients had the only *P. inui*–positive samples, but we identified a few *P. knowlesi*–positive samples among the 71 persons screened at the camp (Appendix Figure 1). The *An. cracens* mosquito was the only *Plasmodium*-positive mosquito we detected. We hypothesize that PMAR0041, PMAR0052, and the *Plasmodium*-positive mosquito were monoinfected with *P. inui* because we found no other *Plasmodium* species in any of them.

## Conclusions 

In experimentally infected humans, patent *P. inui* infections appeared >31 days after infectious mosquito bites (*8*). Similarly, both cases we report show a patent infection ≈30 days after alleged exposure. *P. inui* undergoes a 72-hour erythrocytic cycle, causing quartan fever (*8*). Infection by the quartan *P. inui* could be self-limiting in humans because the parasite was not detected in case-patient PMAR0041 ≈8 months after exposure. Indeed, *P. inui* infections in monkeys are usually low-grade and chronic and can be self-limiting (*9*,*10*). In addition, the *P. inui* OS strain parasite count in experimentally infected humans was <2,520/µL blood. Symptoms were mild, and parasitemia could be submicroscopic or undetectable for certain periods. Antimalarial intervention was deemed unnecessary in these experimental infections (*8*).

Natural human *P. inui* infection seems possible, but because of the very low number of parasites and sharp fluctuations between negative and moderate parasitemia by microscopy (*8*,*10*), previous studies that used less sensitive methods, including standard PCR, were not able to detect it (*11*). We show that nPCR on concentrated, high-volume blood was more sensitive at detecting low-grade infection than standard PCR (*12*), which highlights the need for ultrasensitive detection tools. 

We found 2 forms of *Plasmodium* 18S rRNA genes: the asexual type, which is expressed during the parasite’s asexual life cycle in the vertebrate host; and the sexual type, which is expressed during its sexual life cycle in the mosquito vector. The *Plasmodium*-genus PCR primers we used amplify asexual and sexual 18S rRNA, but the *P. inui*–specific primers only amplify the asexual type, which explains the negative results from the species-specific nPCR despite the positive amplifications in the *Plasmodium*-genus PCR. Nonetheless, successful PCR amplification is compounded by low levels of parasites and the subsequent chance effect that can lead to occasional spurious results, as we experienced.

*P. inui* sporozoites have been found naturally occurring in *An. cracens* mosquitoes (*2*). Other mosquito species from the Leucosphyrus group can transmit *P. inui* naturally (*2*). In addition, laboratory experiments showed *P. inui* adapted to co-indigenous *Anopheles* mosquito species (*13*).

*P. inui* has a wide geographic range in Asia, including southern India, Southeast Asia, and Taiwan (*13*). A surveillance study reported that the prevalence of *P. inui* among wild macaques in Pahang was 66.7% (26/39 macaques sampled); 76.9% of these infections were co-infections with other *Plasmodium* species (*14*). Given the high prevalence of *P. inui* among macaques and natural *Anopheles* mosquito vectors (*2*), humans could be exposed to *P. inui* via vectorborne transmission from infected macaques, particularly at a location where humans, macaque hosts, and mosquito vectors co-exist. Furthermore, studies report that *P. inui* often occurs in co-infections with *P. knowlesi* and *P. cynomolgi* in monkeys and mosquitoes (*2*), and that humans frequently can be exposed to a mix of nonhuman primate malaria sporozoites (*15*). Because human *P. inui* infections can be asymptomatic, *P. inui* could evolve to efficiently infect humans (*2*), especially considering patent human infection can be established by just a few parasites (*8*). Strains from different geographic locations might even exhibit different infection patterns. Investigators should use ultrasensitive methods for epidemiologic and entomological studies of simian malaria transmissions in Malaysia and other countries in malaria elimination efforts.

Appendix Additional information *Plasmodium inui* infections in humans and *Anopheles cracens* mosquito, Malaysia.

## References

[1] Coatney GR, Collins WE, Warren M, Contacos PG. The primate malarias. Washington DC: National Institutes of Health; 1971.

[2] Jeyaprakasam NK, Liew JWK, Low VL, Wan-Sulaiman W-Y, Vythilingam I. *Plasmodium knowlesi* infecting humans in Southeast Asia: What’s next? PLoS Negl Trop Dis. 2020;14:e0008900. 10.1371/journal.pntd.0008900 33382697 PMC7774830

[3] Imwong M, Hanchana S, Malleret B, Rénia L, Day NPJ, Dondorp A, et al. High-throughput ultrasensitive molecular techniques for quantifying low-density malaria parasitemias. J Clin Microbiol. 2014;52:3303–9. 10.1128/JCM.01057-14 24989601 PMC4313154

[4] Singh B, Bobogare A, Cox-Singh J, Snounou G, Abdullah MS, Rahman HA. A genus- and species-specific nested polymerase chain reaction malaria detection assay for epidemiologic studies. Am J Trop Med Hyg. 1999;60:687–92. 10.4269/ajtmh.1999.60.687 10348249

[5] Imwong M, Tanomsing N, Pukrittayakamee S, Day NP, White NJ, Snounou G. Spurious amplification of a *Plasmodium vivax* small-subunit RNA gene by use of primers currently used to detect *P. knowlesi.* J Clin Microbiol. 2009;47:4173–5. 10.1128/JCM.00811-09 19812279 PMC2786678

[6] Lee KS, Divis PCS, Zakaria SK, Matusop A, Julin RA, Conway DJ, et al. *Plasmodium knowlesi*: reservoir hosts and tracking the emergence in humans and macaques. PLoS Pathog. 2011;7:e1002015. 10.1371/journal.ppat.1002015 21490952 PMC3072369

[7] Chua TH, Manin BO, Daim S, Vythilingam I, Drakeley C. Phylogenetic analysis of simian Plasmodium spp. infecting Anopheles balabacensis Baisas in Sabah, Malaysia. PLoS Negl Trop Dis. 2017;11:e0005991. 10.1371/journal.pntd.0005991 28968395 PMC5638607

[8] Coatney GR, Chin W, Contacos PG, King HK. *Plasmodium inui*, a quartan-type malaria parasite of Old World monkeys transmissible to man. J Parasitol. 1966;52:660–3. 10.2307/3276423 5969104

[9] Garnham PCC. The mosquito transmission of *Plasmodium inui* Halberstaedter and Prowazek, and its pre-erythrocytic development in the liver of the rhesus monkey. Trans R Soc Trop Med Hyg. 1951;45:45–52. 10.1016/S0035-9203(51)90524-X 14876765

[10] Schmidt LH, Fradkin R, Harrison J, Rossan RN, Squires W. The course of untreated *Plasmodium inui* infections in the rhesus monkey (*Macaca mulatta*). Am J Trop Med Hyg. 1980;29:158–69. 10.4269/ajtmh.1980.29.158 6768313

[11] Siner A, Liew ST, Kadir KA, Mohamad DSA, Thomas FK, Zulkarnaen M, et al. Absence of *Plasmodium inui* and *Plasmodium cynomolgi*, but detection of *Plasmodium knowlesi* and *Plasmodium vivax* infections in asymptomatic humans in the Betong division of Sarawak, Malaysian Borneo. Malar J. 2017;16:417. 10.1186/s12936-017-2064-9 29041929 PMC5645983

[12] Hofmann NE, Gruenberg M, Nate E, Ura A, Rodriguez-Rodriguez D, Salib M, et al. Assessment of ultra-sensitive malaria diagnosis versus standard molecular diagnostics for malaria elimination: an in-depth molecular community cross-sectional study. Lancet Infect Dis. 2018;18:1108–16. 10.1016/S1473-3099(18)30411-0 30170986

[13] Collins WE, Sullivan JS, Galland GG, Nace D, Williams A, Williams T, et al. Isolates of *Plasmodium inui* adapted to *Macaca mulatta* monkeys and laboratory-reared anopheline mosquitoes for experimental study. J Parasitol. 2007;93:1061–9. 10.1645/GE-1035R.1 18163340

[14] Amir A, Shahari S, Liew JWK, de Silva JR, Khan MB, Lai MY, et al. Natural *Plasmodium* infection in wild macaques of three states in peninsular Malaysia. Acta Trop. 2020;211:105596. 10.1016/j.actatropica.2020.105596 32589995

[15] Maeno Y, Quang NT, Culleton R, Kawai S, Masuda G, Nakazawa S, et al. Humans frequently exposed to a range of non-human primate malaria parasite species through the bites of *Anopheles dirus* mosquitoes in South-central Vietnam. Parasit Vectors. 2015;8:376. 10.1186/s13071-015-0995-y 26178324 PMC4504216

