# Supplementary material for: Natural Plasmodium inui Infections in Humans and Anopheles cracens Mosquito, Malaysia
Source: Emerg Infect Dis. 2021 Oct;27(10):2700–3. doi: 10.3201/eid2710.210412 (PMC8462313; doi:10.3201/eid2710.210412)
Supplement: Appendix — Additional information Plasmodium inui infections in humans and Anopheles cracens mosquito, Malaysia. [file 21-0412-Techapp-s1.pdf]

Article DOI: https://doi.org/10.3201/eid2710.210412

Natural *Plasmodium inui* Infections in Humans and *Anopheles cracens* Mosquito, Malaysia

Appendix

Materials and Methods

High-volume DNA Purification

We used Gentra Puregene Blood Kit (QIAGEN, https://www.qiagen.com) to perform DNA purification by using the following methods. We collected ≤3 mL of blood from each study participant into EDTA blood tubes. We centrifuged blood at 3,000 rpm for 10 min to separate it into distinct layers of plasma, buffy coat, and packed red blood cells. We noted the volume of the whole blood and packed red blood cells. We discarded the plasma, then thoroughly removed the buffy coat. Then, we dispensed 1.5 mL of red blood cell lysis solution into a 2-mL microcentrifuge tube. We added packed red blood cells per 500 µL of whole blood to the tube and mixed by inverting 10 times. We left the mixture to incubate at room temperature for 1 min, with ≥1 inversion during the incubation period. We centrifuged the mixture for 30 s at 13,000 × g. We discarded the supernatant, leaving ≈10 µL of the residual liquid and the DNA pellet. Then, we vigorously vortexed the tube to resuspend the pellet in the residual liquid, after which we added 500 µL of Cell Lysis Solution (QIAGEN) to the tube, and mixed vigorously for 10 s. Next, we pipetted 167 µL of Protein Precipitation Solution (QIAGEN) into the tube, then mixed vigorously for 20 s at high speed. We centrifuged the mixture for 1 min at 13,000 × g before transferring the supernatant into a 1.5 mL tube containing 500 µL of isopropanol. We gently inverted the mixture 50 times until the DNA was visible as threads or a clump. Then, we centrifuged the mixture for 1 min at 13,000 × g. We discarded the supernatant before adding 500 µL of 70% ethanol to wash the DNA pellet. We centrifuged the tube for 1 min at 13,000 × g then left the DNA pellet to air dry for 5 min. Then, we added 50 µL of DNA Hydration Solution (QIAGEN) to the tube and vortexed for 5 s at medium speed to mix. We incubated the mixture at

65°C for 5 min to dissolve the DNA pellet before incubating at room temperature overnight with gentle shaking by using a rotator. Finally, we lyophilized or concentrated the DNA solution to only 5 μL. Using this protocol, we concentrated DNA from 500 μL of whole blood into 5 μL of solution.

High-Volume Nested PCR to Detect *Plasmodium* 18S rRNA Gene

By using the DNA extracted in the previous step, we performed *Plasmodium* genus-specific PCR using primers rPLU1 and rPLU5 for the first PCR reaction, then primers rPLU3 and rPLU4 for the nested PCR (nPCR) reaction, as previously described (*1*). We used 2 μL of DNA in a 25 μL PCR reaction containing 1 U of GoTaq G2 Flexi DNA polymerase (Promega, https://www.promega.com), 4 mmol $MgCl_2$, 0.2 mmol of each dNTP, and 0.25 μmol of primers. We used the following cycling parameter for the first PCR reaction: initial denaturation at 94°C for 4 min, then 30 cycles of 94°C for 1 min, 55°C for 1 min, 72°C for 1 min, and a final extension at 72°C for 8 min. Next, we used 3 μL of the PCR product from the first PCR reaction in the nPCR reaction with the same PCR mixture as mentioned above. We used the following cycling parameter for the second nPCR reaction: initial denaturation at 94°C for 4 min, then 30 cycles of 94°C for 30 s, 58°C for 1 min, 72°C for 1 min, and a final extension at 72°C for 10 min. We incorporated negative controls in the assays by using DNA from blood of a healthy donor, purified by using the same protocol, as a template in the PCR reaction. We used no-template controls in all PCR reactions by using ddH_2O in place of DNA templates in the PCR reactions.

We conducted similar species-specific nPCR to detect *P. falciparum*, *P. vivax*, *P. malariae* (*1*), *P. knowlesi* (*2*), *P. ovale* (*3*), and *P. cynomolgi* (*4*), except we used 35 cycles for the nest 2 PCRs specific for *P. inui* (*4*) at an annealing temperature of 55°C. We cloned positive PCR products into pGEM-T vector (Promega) and sequenced positive clones.

DNA Extraction and nPCR to *Plasmodium* Species in *Anopheles* Mosquito

We extracted genomic DNA from the salivary gland or head and thorax of mosquitoes by using the DNeasy tissue kit (QIAGEN) according to the manufacturer's protocol. We performed nPCR assay targeting the *Plasmodium* small subunit ribosomal RNA (18S rRNA) gene to identify human malaria parasites (*P. falciparum*, *P. malariae*, *P. ovale*, and *P. vivax*) and simian malaria parasites (*P. coatneyi*, *P. cynomolgi*, *P. fieldi*, *P. inui* and *P. knowlesi*) by using genus-

specific primers rPLU1 and rPLU5 for the nest 1 amplification (*1*), then species-specific primers in the nest 2 amplification (*1–4*).

We performed the first PCR assay in a final volume of 50 μL containing 5 μL of DNA template, 1× GoTaq Green reaction buffer (Promega), 3 mmol $MgCl_2$ (Promega), 0.2 μmol of dNTP mixture (Promega), 0.25 μmole each of forward (rPLU1) and reverse (rPLU5) primers and 1.25 U of GoTaq G2 Flexi DNA polymerase (Promega). We used the following cycling parameter for the first PCR: initial denaturation at 94°C for 4 min, then 35 cycles of 94°C for 30 s, 55°C for 1 min, 72°C for 1 min, and a final extension at 72°C for 10 min. Then for each 20 μL of nest 2 PCR amplification, we used 3 μL of PCR product from the first PCR reaction as DNA template. We used identical concentrations of reagents in the nest 2 amplifications to those used in the nest 1 reactions, except the final concentration of the GoTaq G2 Flexi DNA polymerase (Promega) was 1 U. We also used identical PCR conditions as in the first PCR amplification, except for the annealing temperatures; *P. knowlesi* and *P. inui* were annealed at 58°C; *P. coatneyi* and *P. cynomolgi* were annealed at 60°C; and *P. fieldi* was annealed at 63°C. We also used 4 μL of PCR product from the first PCR reaction as a DNA template to detect human-specific malaria parasites *P. falciparum*, *P. vivax*, *P. malariae*, and *P. ovale* by using primers and protocols described previously (*1*). We analyzed the amplification products by using 1.5% agarose gel electrophoresis.

18S rRNA Sequencing of *P. inui* from *Anopheles cracens* Mosquito

We subjected the *P. inui*–positive mosquito sample to another nPCR targeting a larger fragment of the 18S rRNA gene. We performed PCR amplification reaction for the first PCR assay in a final volume of 50 μL containing 5 μL of DNA template, 1× GoTaq Long PCR master mix (Promega), and 0.25 μmol each of forward (rPLU1) and reverse (rPLU5) primers. We used the following cycling parameter for the first PCR reaction: initial denaturation at 94°C for 4 min, followed by 40 cycles of 94°C for 30 s, 55°C for 1 min, 72°C for 1.5 min, and a final extension at 72°C for 10 min. We performed PCR amplification reaction for the nest 2 assay by using a universal forward primer UMSF (*5*) combined with species-specific primer INAR3 (*4*). We performed the nest 2 PCR assay in a final volume of 25 μL containing 3 μL of DNA template from nest 1, 1× GoTaq Green Reaction Buffer (Promega), 2 mmol $MgCl_2$ (Promega), 0.2 μmol of dNTP mixture (Promega), 0.4 μmol each of the forward and reverse primer, and 1.5 U of GoTaq G2 Flexi DNA Polymerase (Promega). We used a similar cycling parameter for nest 2 to

the first PCR reaction, except the duration for the first denaturation was 5 min, then 35 cycles of 1 min each of denaturation, annealing, and extension. We analyzed the amplified products by using 1.5% agarose gel electrophoresis. We observed the correct band in the gel which we excised and sent for sequencing.

References

1. Singh B, Bobogare A, Cox-Singh J, Snounou G, Abdullah MS, Rahman HA. A genus- and species-specific nested polymerase chain reaction malaria detection assay for epidemiologic studies. Am J Trop Med Hyg. 1999;60:687–92. PubMed https://doi.org/10.4269/ajtmh.1999.60.687

2. Imwong M, Tanomsing N, Pukrittayakamee S, Day NP, White NJ, Snounou G. Spurious amplification of a *Plasmodium vivax* small-subunit RNA gene by use of primers currently used to detect *P. knowlesi.* J Clin Microbiol. 2009;47:4173–5. PubMed https://doi.org/10.1128/JCM.00811-09

3. Fuehrer HP, Stadler MT, Buczolich K, Bloeschl I, Noedl H. Two techniques for simultaneous identification of *Plasmodium ovale curtisi* and *Plasmodium ovale wallikeri* by use of the small-subunit rRNA gene. J Clin Microbiol. 2012;50:4100–2. PubMed https://doi.org/10.1128/JCM.02180-12

4. Lee KS, Divis PC, Zakaria SK, Matusop A, Julin RA, Conway DJ, et al. *Plasmodium knowlesi*: reservoir hosts and tracking the emergence in humans and macaques. PLoS Pathog. 2011;7:e1002015. PubMed https://doi.org/10.1371/journal.ppat.1002015

5. Chua TH, Manin BO, Daim S, Vythilingam I, Drakeley C. Phylogenetic analysis of simian *Plasmodium* spp. infecting *Anopheles balabacensis* Baisas in Sabah, Malaysia. PLoS Negl Trop Dis. 2017;11:e0005991. PubMed https://doi.org/10.1371/journal.pntd.0005991

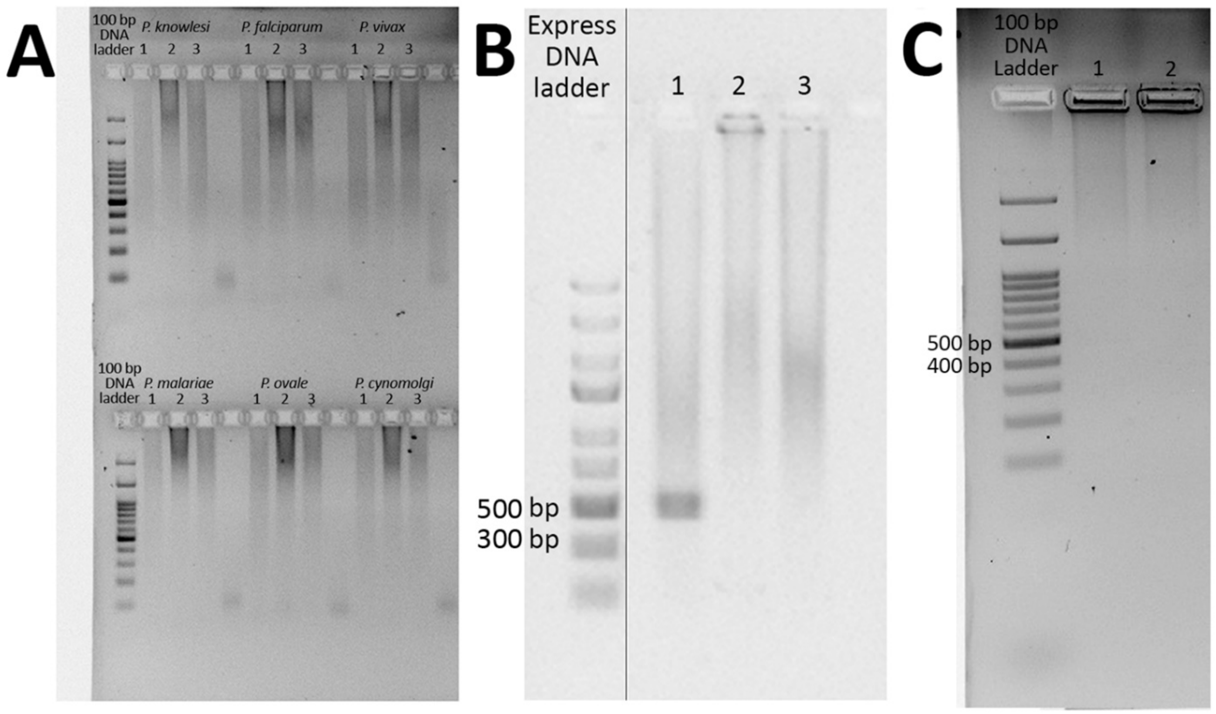

Appendix Figure 1. *Plasmodium* genus-specific nested PCR results of 2 cases of human *Plasmodium inui* infection, Malaysia. Samples were subjected to electrophoresis on a 1.5% agarose gel. A) Results for case PMAR0041. Lane 1, 100 bp DNA ladder; lane 2, case PMAR0041; lanes 3 and 4, negative control; lane 5, *P. knowlesi*–positive control; lane 6, no-template control. B) Results for case PMAR0052. Lane 1, 100 bp DNA ladder; lane 2, case PMAR0052; lane 3, negative control; lane 4, no-template control. The unlabeled lanes represent *P. inui*-negative samples.

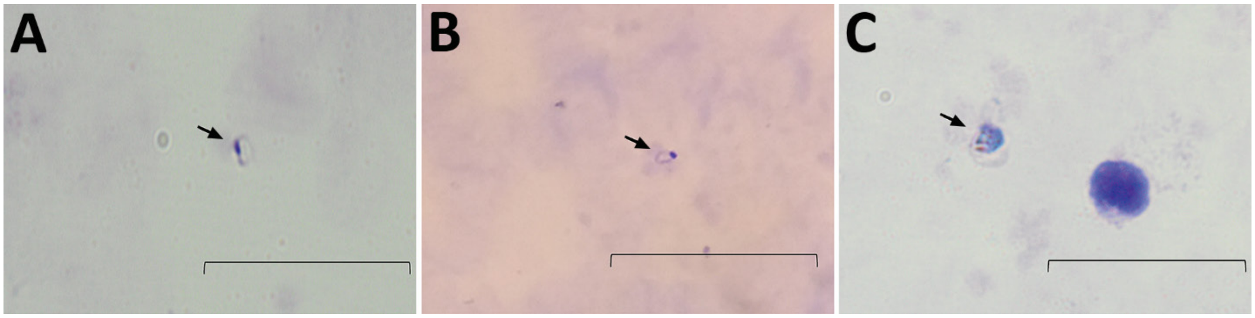

Appendix Figure 2. CLUSTAL OMEGA alignment of *Plasmodium inui* 18S rRNA sequences from human cases PMAR0041 and PMAR0052 and part of the *P. inui* sequence obtained from an *Anopheles cracens* mosquito collected in the same area as the 2 human cases. GenBank accession numbers are in parentheses. The asterisks indicate identical nucleotides in all the 3 sequences at that position.
